# Supplementary figures and images for: Clinical and social determinants of health features of SARS-CoV-2 infection among Black and Caribbean Hispanic patients with heart failure: The SCAN-MP Study
Source: PLoS One. 2023 Mar 30;18(3):e0283730. doi: 10.1371/journal.pone.0283730 (PMC10062570; doi:10.1371/journal.pone.0283730)

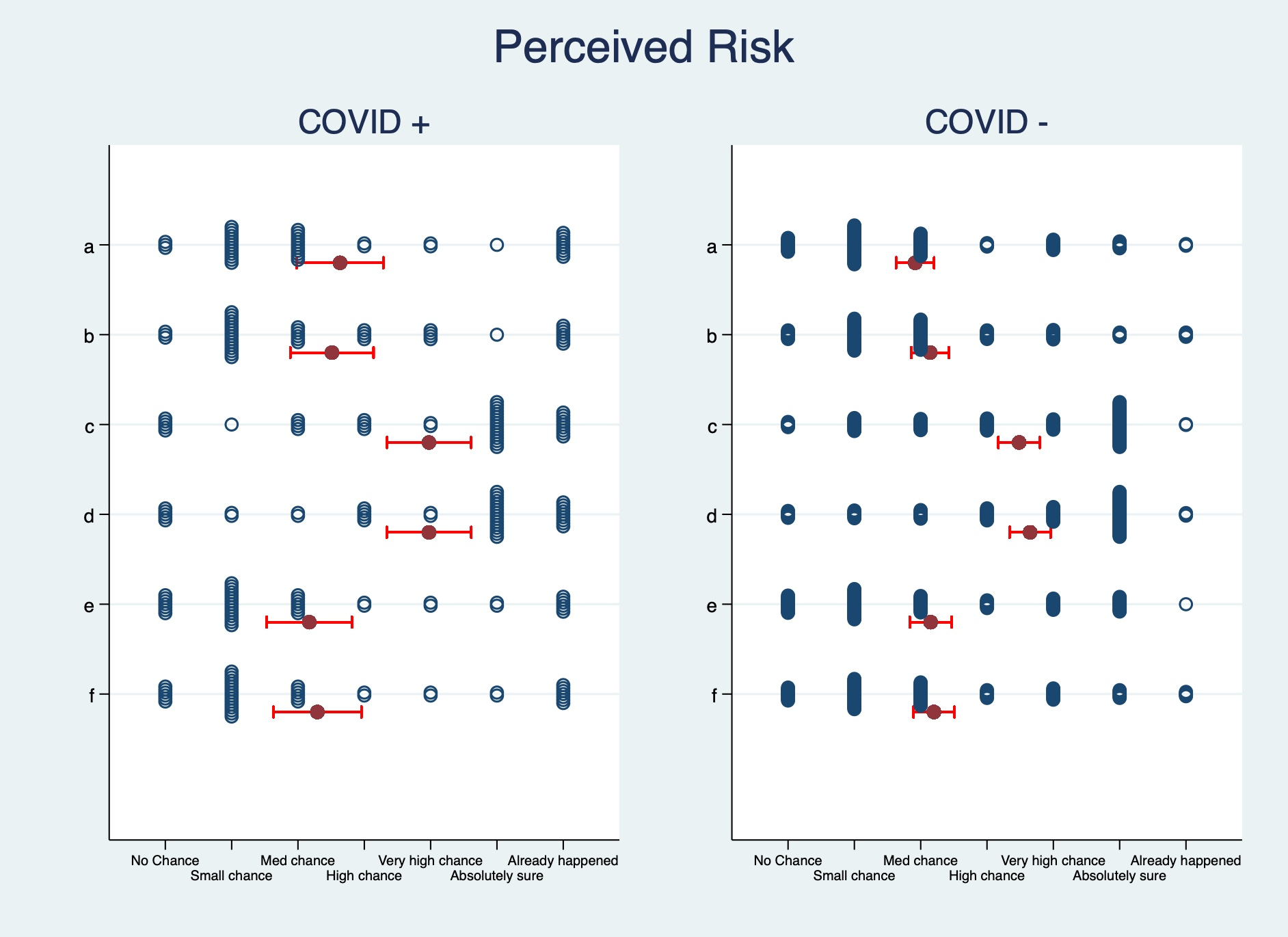

Supplement: S1 Fig — KEY: (a)”You will be infected” (b)“Someone in your direct environment (family, friends, or colleagues) will be infected” (c)”You will have to go to the hospital if you get infected” (d)”You will have to go into quarantine” (e)”You will get infected and you will infect someone else” (f)”Someone in your direct circle of people (family, friends, colleagues) will become ill or die”. The hollow circles represent subject selection of answers. Red circle represents the average(mean) response, with the horizontal line representing the 95% confidence interval. (TIF) [file pone.0283730.s001.tif]

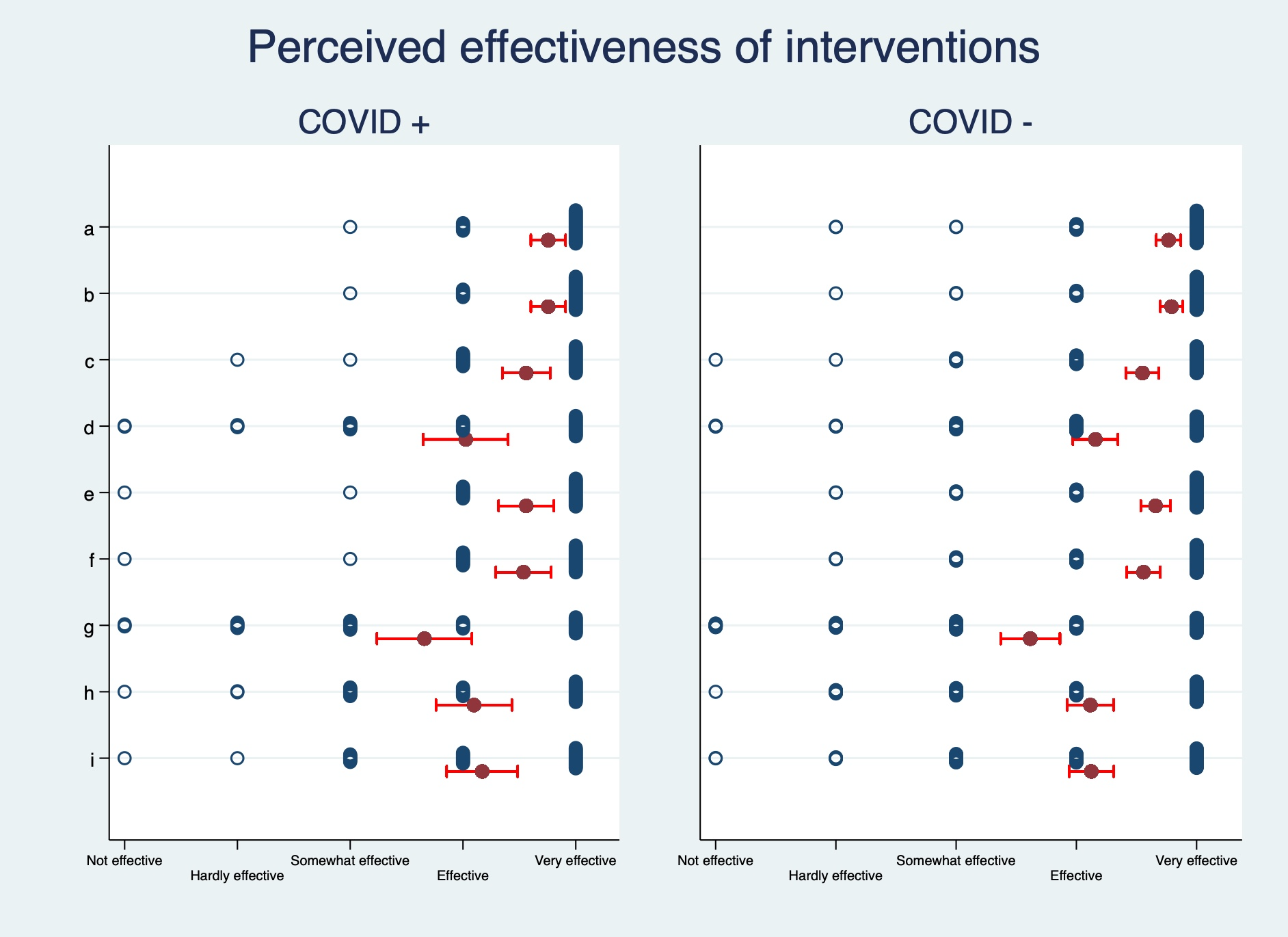

Supplement: S2 Fig — KEY: (a)”Wearing a mask” (b)”Washing your hands with soap or using hand sanitizer frequently” (c)”Seeing a health care provider if you feel sick” (d) “Seeing a health care provider if you feel healthy but worry that you were exposed” (e)”Avoiding public spaces, gatherings, and crowds” (f) “Avoiding contact with people who could be high-risk” (g) “Avoiding hospitals and clinics” (h) “Avoiding restaurants” (i)”Avoiding public transit”. The hollow circles represent subject selection of answers. Red circle represents the average(mean) response, with the horizontal line representing the 95% confidence interval. (TIF) [file pone.0283730.s002.tif]

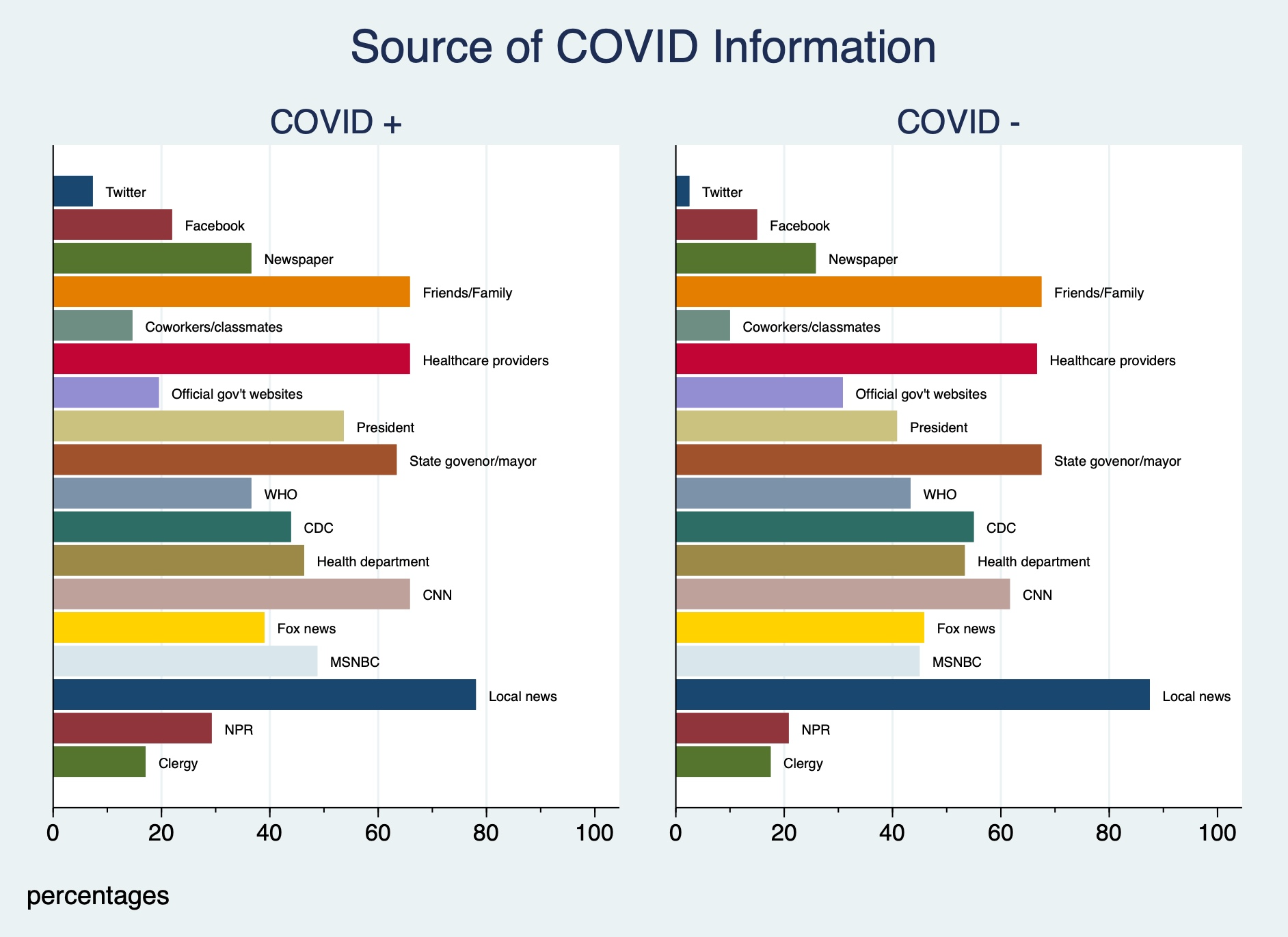

Supplement: S3 Fig — (TIF) [file pone.0283730.s003.tif]
