# Supplementary material for: Clinical and social determinants of health features of SARS-CoV-2 infection among Black and Caribbean Hispanic patients with heart failure: The SCAN-MP Study
Source: PLoS One. 2023 Mar 30;18(3):e0283730. doi: 10.1371/journal.pone.0283730 (PMC10062570; doi:10.1371/journal.pone.0283730)
Supplement: S1 File — (DOCX) [file pone.0283730.s004.docx]

**Supplemental Material:**

**COVID-19 Questionnaire (attached)**

**References for assessments:**

Health Literacy – Weiss BD, Mays MZ, Martz W, Castro KM, DeWalt DA, Pignone MP, Mockbee J, Hale FA. Quick assessment of literacy in primary care: the newest vital sign. The Annals of Family Medicine. 2005 Nov 1;3(6):514-22.

Trust in Providers – Anderson LA, Dedrick RF. Development of the Trust in Physician scale: a measure to assess interpersonal trust in patient-physician relationships. Psychological reports. 1990 Dec;67(3_suppl):1091-100.

Perceived discrimination – Sternthal, M., Slopen, N., Williams, D.R. “Racial Disparities in Health: How Much Does Stress Really Matter?” Du Bois Review, 2011; 8(1): 95-113.

**Supplemental Figure 1**

Figure 8: Perceived Risk. KEY: (a)”You will be infected” (b)“Someone in your direct environment (family, friends, or colleagues) will be infected” (c)”You will have to go to the hospital if you get infected” (d)”You will have to go into quarantine” (e)”You will get infected and you will infect someone else ” (f)”Someone in your direct circle of people (family, friends, colleagues) will become ill or die”. The hollow circles represent subject selection of answers. Red circle represents the average(mean) response, with the horizontal line representing the 95% confidence interval.


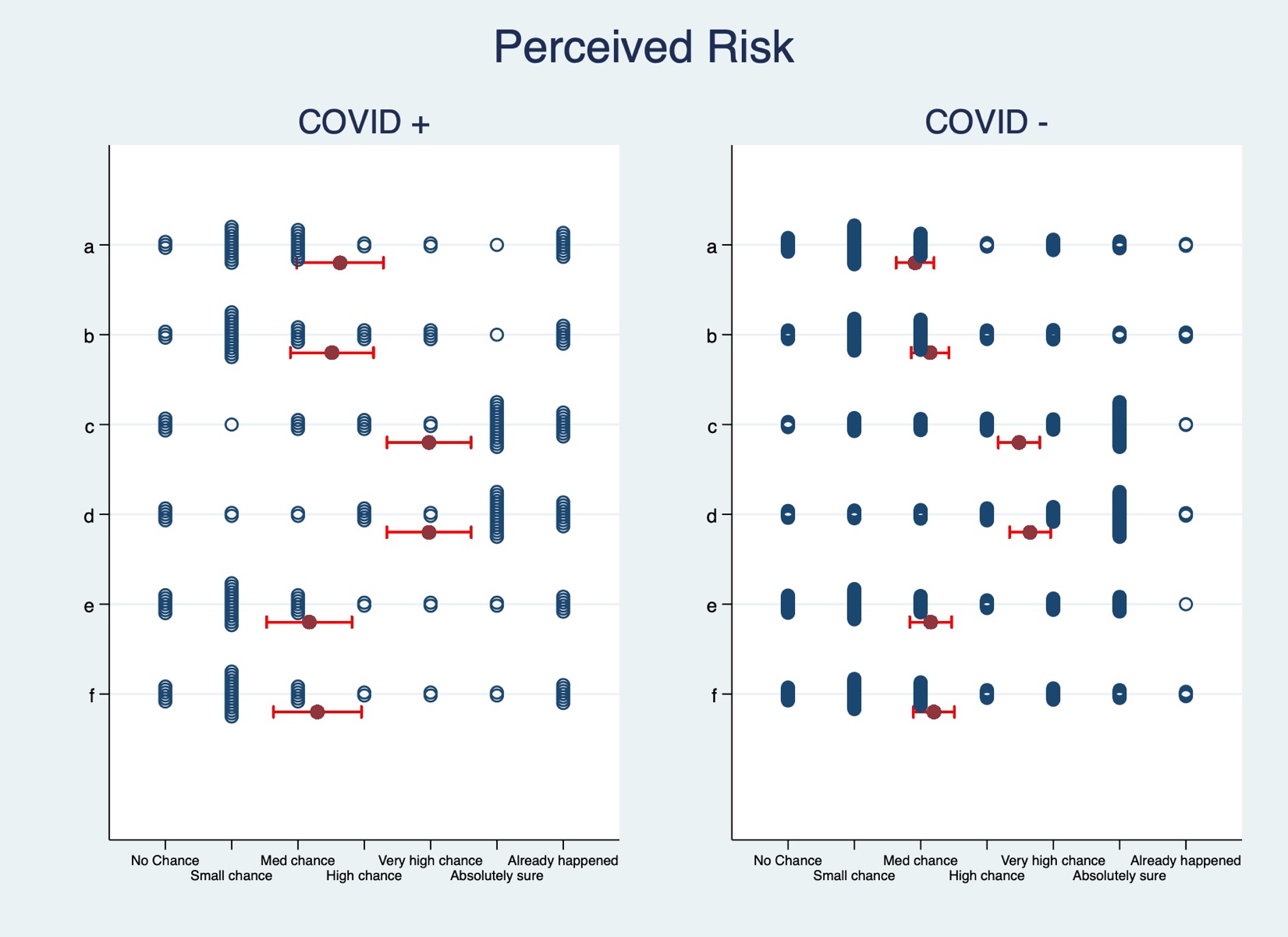


**Supplemental Figure 2**

Perceived effectiveness of interventions. KEY: (a)”Wearing a mask” (b)”Washing your hands with soap or using hand sanitizer frequently” (c)”Seeing a health care provider if you feel sick” (d) “Seeing a health care provider if you feel healthy but worry that you were exposed” (e) ”Avoiding public spaces, gatherings, and crowds” (f) “Avoiding contact with people who could be high-risk” (g) “Avoiding hospitals and clinics” (h) “Avoiding restaurants” (i)”Avoiding public transit”. The hollow circles represent subject selection of answers. Red circle represents the average(mean) response, with the horizontal line representing the 95% confidence interval.


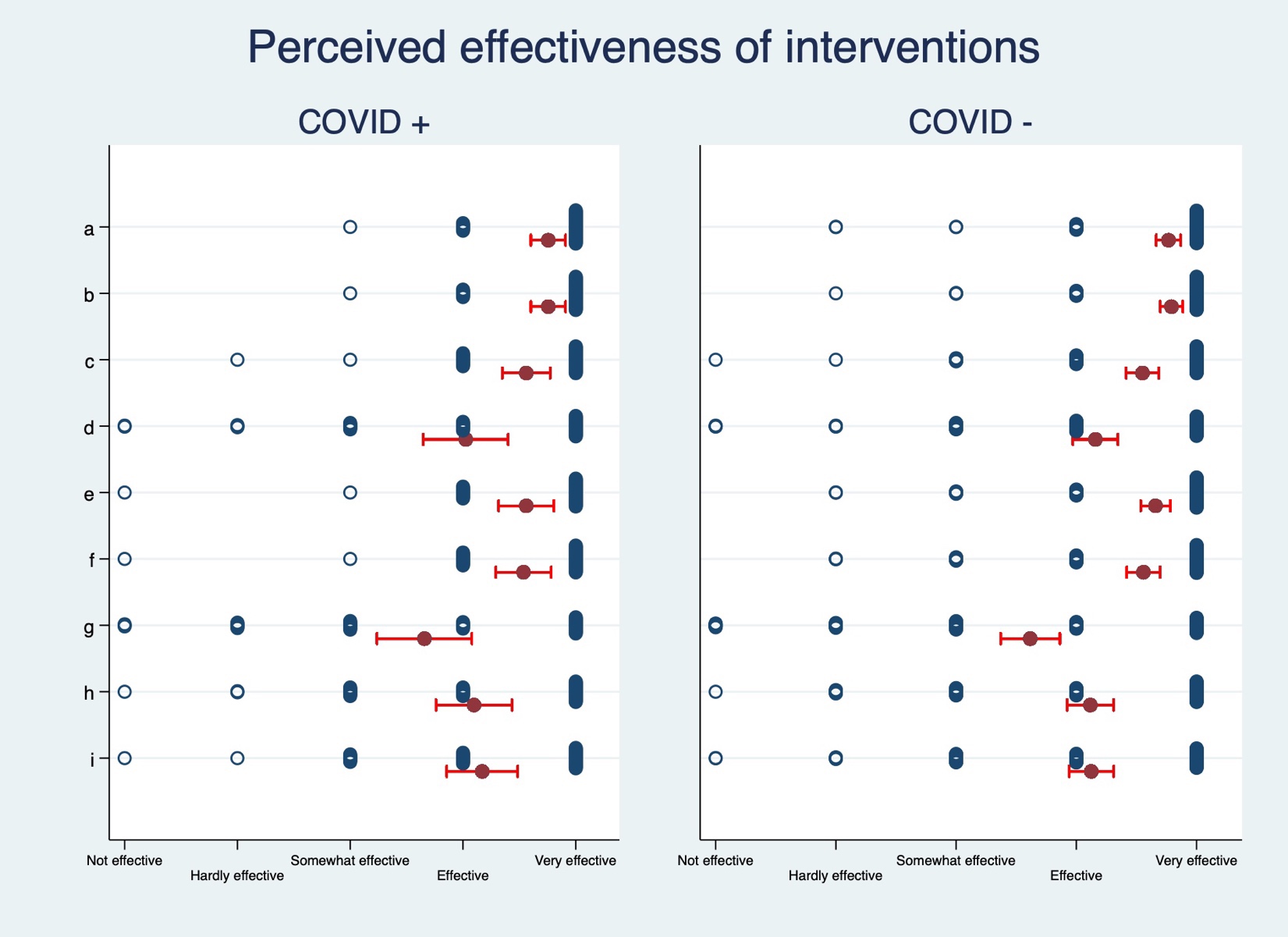


**Supplemental Figure 3**


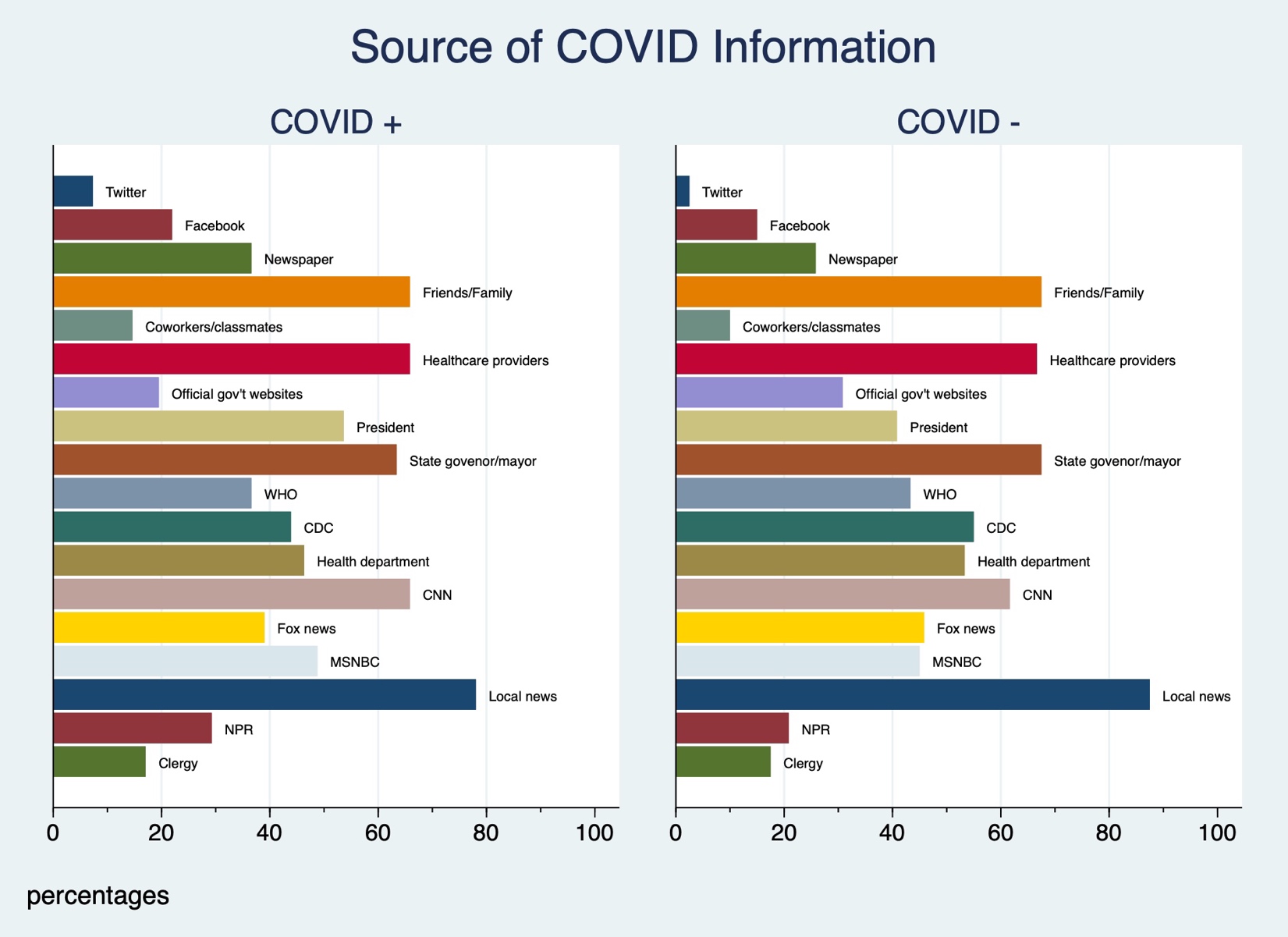


**Supplemental Table 1**: Test positivity rates by site. This table shows similar information to Figure 2

|  | Boston | | | NYC | | |
| --- | --- | --- | --- | --- | --- | --- |
| Month, Year | Total Tested | Positive | Positive Rate | Total Tested | Positive | Positive Rate |
| Dec/19 | 6 | 0 | 0% | 2 | 0 | 0% |
| Jan/20 | 2 | 0 | 0% | 9 | 4 | 44% |
| Feb/20 | 5 | 0 | 0% | 6 | 2 | 33% |
| Mar/20 | 2 | 1 | 50% | 1 | 0 | 0% |
| Apr/20 | COVID Pause in Recruitment | | | | | |
| May/20 |  |  |  |  |  |  |
| Jun/20 |  |  |  |  |  |  |
| Jul/20 | 0 | 0 | 0% | 5 | 2 | 40% |
| Aug/20 | 1 | 0 | 0% | 5 | 1 | 20% |
| Sep/20 | 4 | 0 | 0% | 5 | 2 | 40% |
| Oct/20 | 3 | 1 | 33% | 6 | 2 | 33% |
| Nov/20 | 4 | 0 | 0% | 8 | 4 | 50% |
| Dec/20 | 5 | 2 | 40% | 0 | 0 | 0% |
| Jan/21 | 4 | 0 | 0% | 5 | 3 | 60% |
| Feb/21 | 4 | 1 | 25% | 3 | 0 | 0% |
| Mar/21 | 10 | 1 | 10% | 5 | 3 | 60% |
| Apr/21 | 7 | 0 | 0% | 5 | 2 | 40% |
| May/21 | 1 | 1 | 100% | 7 | 2 | 29% |
| Jun/21 | 6 | 1 | 17% | 7 | 3 | 43% |
| Jul/21 | 5 | 1 | 20% | 8 | 3 | 38% |
| Aug/21 | 0 | 0 | 0% | 7 | 2 | 29% |
| Sep/21 | 3 | 1 | 33% | 9 | 5 | 56% |
| Oct/21 | 0 | 0 | 0% | 4 | 0 | 0% |
