# Supplementary material for: Clinical and social determinants of health features of SARS-CoV-2 infection among Black and Caribbean Hispanic patients with heart failure: The SCAN-MP Study
Source: PLoS One. 2023 Mar 30;18(3):e0283730. doi: 10.1371/journal.pone.0283730 (PMC10062570; doi:10.1371/journal.pone.0283730)
Supplement: Attachment — Submitted filename: Response to Reviewers3JanFinal.docx [file pone.0283730.s005.docx]

**Responses to reviewers’ comments**

**Title: Clinical and social determinants of health features of SARS-CoV-2 infection among Black and Caribbean Hispanic patients with heart failure: The SCAN-MP Study**

**Manuscript ID: PONE-D-22-19779**

We thank the reviewers for their valuable feedback and have modified our manuscript accordingly. We have also edited the manuscript to conform to Journal style as requested.

**Reviewer #2:**

**1) Clinically used classic myocardial injury indicators, which can effectively reflect the myocardial injury of patents, could be added to the analysis of the outcome of SARS-CoV-2 infection patients with heart failure.**

We agree with the reviewer’s point, but unfortunately, we do not have myocardial injury markers available at this time. Injury markers, including high sensitivity troponin I and T, will be measured at the completion of the entire SCAN-MP study in 2024 or 2025 in batch through a contract with Abbott Laboratories. We might add that injury markers are likely to be abnormal given that these patients have chronic heart failure that commonly is associated with low level elevations in high sensitivity markers.

**2) NYHA class changes before and after infection should be described to demonstrate no increased mortality or hospitalizations among those infected with SARS-CoV-2.**

We agree that having pre and post infection NYHA class could be useful, but unfortunately the design of our study is a baseline formal assessment of NYHA class (as we have presented) with limited phone followup. We do not have information regarding serial change in NYHA class.

**3) Another limitation is the absence of the number of the severe and non-severe COVID-19, which may have affected outcomes**.

We agree that this is an important limitation of our study and have clarified this in the limitation section of our manuscript with the following:

“Additionally, patients were only questioned about hospitalizations 6 months prior to the start of the study, but for anyone enrolled at or after July of 2020 (6 months after the start of the pandemic), COVID related hospitalizations could have been missed. *Additionally, survival bias attenuates the effect size of hospitalization and death*.”

Thank you for the opportunity to respond to these thoughtful comments.

**Reviewer #3**

References have been added as requested – new references 19 and 20.
